# Hyperbaric effects on heart rate in professional SCUBA divers in thermal water

**DOI:** 10.3389/fspor.2024.1429732

**Published:** 2024-09-30

**Authors:** Luigi Fattorini, Angelo Rodio, Tommaso Di Libero, Cristian Ieno, Giovanna Tranfo, Daniela Pigini, Alessandro Pinto, Enrico Marchetti

**Affiliations:** ^1^ Department of Physiology and Pharmacology “Vittorio Erspamer,” Sapienza University of Rome, Rome, Italy; ^2^ Department of Human Sciences, Society and Health University of Cassino and Southern Lazio, Cassino, Italy; ^3^ Department of Occupational Medicine, Epidemiology and Hygiene, INAIL, Rome, Italy; ^4^ Experimental Medicine Department, Medical Pathophysiology, Food Science and Endocrinology Section, Food Science, Sapienza University of Rome, Rome, Italy

**Keywords:** bradycardia, dive response, thermoregulation, diving physiology, organ perfusion, venous return

## Abstract

**Introduction:**

Diving in SCUBA modality modifies human physiology in many ways. These modifications have been studied since Paul Bert in a seminal work. This area of research is very sensible to technological development. At now, it is possible to record heart rate (HR) continuously while diving. The study of HR changes in SCUBA diving at different depths in a constant temperature of thermal water is the objective of the present paper.

**Methods:**

18 healthy subjects were enrolled and HR was recorded while SCUBA diving in thermal water at a constant temperature of 33.6^∘^ C in the deepest Italian pool at Montegrotto (Padova, Italy). Three depths were investigated: −20, −30 and −40 meters. The HR has been recorded with a Galileo SOL diving computer. The dive was subdivided into three phases: descent (DSC), steady on depth (STD), post–dive (RSF), and average HR was evaluated in each phase. Moreover, considering the DSC and STD time duration, a statistical linear regression of HR and relative parameters, intercept and slope, were here assessed.

**Results:**

In STD phase, HR slope obtained by regression decreased with depth. A significant difference was found between the slope during STD at −20 vs. −40 m (*p* ≤ 0.05).

**Discussion:**

Present results emphasized different HR physiological adjustments among diving phases. Firstly, during the DSC, a rapid HR decrease is recognized as probably due to a vagal response; secondly, at STD, the inward blood redistribution requires another physiological adjustment. This latter is depth-dependent because of a reduction of cardiac variability. Present data highlight the important cardiac stress need to counteract the diving activity.

## 1 Introduction

Hyperbaric worker exposure is rapidly increasing in Italy and, in general, worldwide. This is due to the increase in the use of tunnel boring machines, which are more economical than other technologies, extractive activities, harbor rigging, and sport-recreational diving. In this background, self-contained underwater breathing apparatus (SCUBA) diving is one of the most frequent working activities that take place along coastal regions, lakes, and waterways and represents one of the most stressful physiological challenges for the human body due to its placement in such an extraordinary environment. Indeed, diving conditions can vary greatly due to different environmental conditions, water densities, altitudes, depths, and temperatures. Among others, diving into cold water induces several effects on the body and triggers compensatory physiological responses; one of the most important is counteracting the rapid heat loss to maintain the core temperature as the human body cools faster in water than in air at the same temperature (1–3). As is well known, body temperature control involves several physiological mechanisms that mainly concern blood flow and a redistribution toward the innermost body and are activated through receptor stimulation (4, 5). Other than thermoregulation, three main reflex responses have been described during diving activity known as “dive response” due to hyperbarism: (i) the bradycardia reflex induced by the vagus nerve for the action of the glomus caroticum, (ii) the redirection of blood flow from peripheral to central organs through the vasoconstriction of selected vascular beds, and (iii) a decrease in metabolism (6–10). Moreover, it is reported that diving causes an evident heart rate (HR) slowing even if during cold-water immersion the HR is increased by facial and brain cooling, which is more effective in experienced divers (11–16). In addition, central factors influence HR changes, as well as emotional ones, immediately before diving, inducing tachycardia (6, 9). Furthermore, some studies have described the increased partial pressure of oxygen, gas density, hydrostatic pressure, and increased gas tensions of nitrogen as possible causes of hyperbaric-induced bradycardia (17–20). As evident, all possible mechanisms, central or local, involved during diving influence HR both directly and indirectly. Moreover, HR changes are related to blood flow and tissue perfusion regulation involving stroke volume and systemic venous return other than hormonal and metabolic control. These adjustments adopted during diving activities appear to be oxygen-conserving mechanisms coordinated through an arterial-baroreflex-mediated mechanism and occur during dry and wet hyperbaric exposure (6, 19, 21, 22). While diving in open water (lake, sea, or ocean), the thermoregulation results are a confounding factor in assessing the sole hyperbaric effects on cardiac adjustments because the water temperature is inversely correlated to depth, and cold influences HR in a variable way with depth. Given all of the above, this study aimed to assess cardiac response in hyperbaric wet conditions at several maximal depths (MDs) in thermal water at a constant temperature (33.6^∘^C) with SCUBA.

## 2 Materials and methods

### 2.1 Study setting and protocol

#### 2.1.1 Study group

The study group consisted of 18 volunteers (15 males and 3 females) who were experienced professional divers and nonsmokers aged 48 + 7 years, with a height of 171 + 12 cm and weight of 76 + 12 kg (mean + SD). Divers physiological and anthropometric characteristics, including a BMI of (26.2 ± 3) and Fat Mass (FM), (20.6 ± 8.3), are shown in Table 1. The subjects were divided into three different experimental sessions: April 2023, August 2023, and April 2024, with six subjects in each session. From the 18 original participants, only 17 completed the experimental sessions (due to technical issues). The inclusion criteria required subjects to have medical diving approval, no drugs active in the cardiovascular system, no smoking habit, the absence of cardiovascular diseases, and at least 5 years of SCUBA diving experience. Measures were obtained for subjects who were carrying out routine safety activities for pool users; therefore, there was no need to request other specific activities. The Ethics Committee of the “LAZIO 2” ASL Roma 2 approved this study (N.0207553/2022), which adhered to the Declaration of Helsinki and followed the International Code of Ethics for Occupational Health Professionals (International Committee of Occupational Health, 2014) also by Istituto Nazionale Assicurazione contro gli Infortuni sul Lavoro. All enrolled participants provided written informed consent voluntarily, after being informed of all the aspects of the project that were relevant to the subject’s decision to participate.

**Table 1:** Divers’ physiological and anthropometrical characteristics.

Divers’ characteristics	Mean	SD
Age (y)	48	7
Height (m)	1.71	0.12
Weight (kg)	76	12
HRrest (bpm min^−1^)	82	8
HRpeak (bpm min^−1^)	164	4
VO2max (ml/kg/min)	30	5
VT1% (respect ${\mathrm{VO}}_{2}$max)	57	4
VT2% (respect ${\mathrm{VO}}_{2}$max)	68	3

#### 2.1.2 Diving protocol and immersion conditions

The experimental sessions were performed in the Y-40 indoor diving pool (Montegrotto Terme, Padova, Italy), at a constant temperature of 33.6^∘^C (thermal water). All experimental sessions were performed in the morning and ended before lunch. All divers were based at the same hotel adjacent to the pool and had meals and carbohydrates at the same time (7:30 a.m.). Scuba divers wore identical wetsuits to equalize the temperature exposure. The dive profile was recorded by an individual dive computer (Galileo Sol, Scubapro Uwatec, California, USA) that recorded depth, duration, HR, tank air pressure, and water temperature only in the wet phase (23). In both sessions, each subject performed three experimental dives on three consecutive days, at 9.00 a.m., at different MDs: 20, 30, and 40 m, remaining at the bottom for 30 min without moving. MDs were randomized to avoid physiological adaptive phenomena. The buddy system implied that two divers had the same MD each day. Decompression procedures were selected from a dive computer. For teams diving at 20 and 30 m MD, there were only 3 min at 3 MD stops. The diving team at 40 MD had to stop at 9 MD for 3 min, at 6 MD for 10 min, and at 3 MD for 30 min. Descent/ascent rates were chosen in accordance with the US Navy Diving Manual rev. 7, 2016: 20 and 10 m/min, respectively. The breathed gas was atmospheric compressed air at 20.3 MPa. Divers maintained depth level with the aid of a buoyancy compensator jacket. All divers wore a wetsuit that covered only the body, a jacket that held the 15 L tank, and a weighting belt, in addition to a mask and fins. Safety was accurate; SCUBA divers had a Galileo computer for gauging depth, dive duration, and safety stops, in addition to a second regulator for redundancy. At the safety stop depth of 3 MD, there were two tanks with double regulators, and at the bottom of the pool (40 MD), there were two tanks with double regulators each. An instructor dived with the subjects to obtain early warnings of eventual problems. The medical board controlled dietary intake during all the experimental sessions and during the week before. To control for circadian effects, for every subject, all experimental sessions were carried out at the same time each day in the morning. There were safety briefings before and after the dive each day. In addition, the theoretical maximum HR (HRpeak) was determined using the standard formula outlined in the literature, which involved subtracting the individual’s age from 220 (24).

### 2.2 Physiological profile assessment

#### 2.2.1 Anthropometric parameters and body composition analysis (BCA)

The anthropometric parameters were acquired according to the standardized procedures described in the Anthropometric Standardization Reference Manual (25): body weight and height were measured respectively to the nearest 0.1 kg using a standard column body scale (SECA, Hamburg, Germany) and to the closest 0.1 cm using a rigid stadiometer (SECA, Hamburg, Germany). Waist circumference was measured to the nearest 0.1 cm using an anthropometric tape. Bioelectrical impedance analysis was applied to estimate body composition, following standardized nutrition procedures (26), using a NUTRILAB device (AKERN Bioresearch SRL, Pontassieve, Florence, Italy).

#### 2.2.2 Diving phases

**Figure 1:**
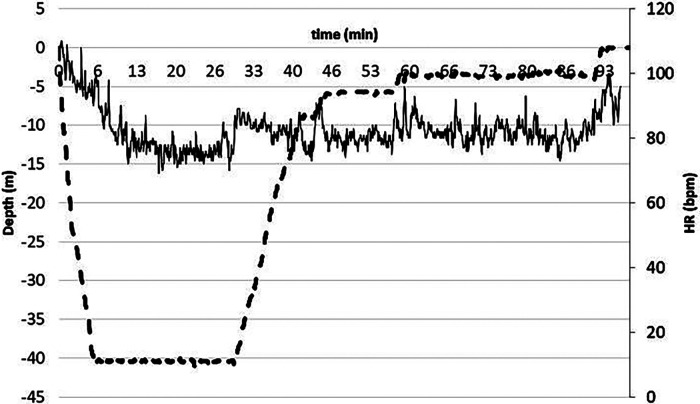
Dotted line represents the dive profile i.e. the diving quote second by second, while continuous the HR. As evident, HR was remarkable high during the pre-dive and decreased rapidly during the descent phase, then decreased slightly during the steady phase, and finally returned to the pre-dive values during resurfacing. Note the decompression stops before resurfacing.

The diving profile was divided as follows: dive from 0 to target depth at a descending speed of approximately 20 m/min [descent phase (DSC)]; 30 min in a relaxed condition at depth [subjects had to avoid moving as much as possible; steady on depth phase (STD)]; 3 min after resurfacing [post-dive phase (RSF)]. As an example, at 40 MD, see Figure 1.

#### 2.2.3 Hearth rate measurements

**Figure 2:**
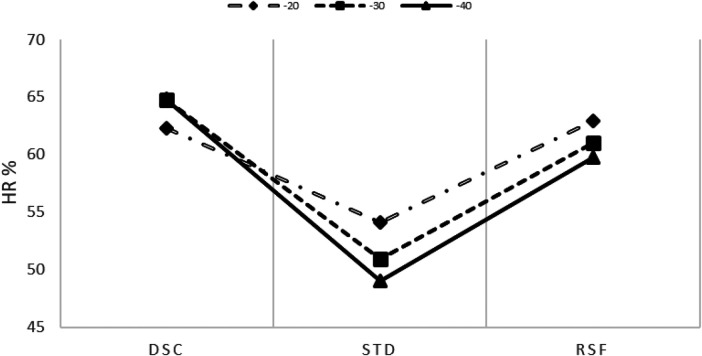
$\bar{HR}$ % (scaled by the HR peak) trend at all depths (−20, −30, and −40 m) during the descent (DSC) and dive (STD), and after resurfacing (RSF). The intercept and slope parameters obtained through linear regression analysis showed no differences in DSC between MDs (p≤0.05).

HR was recorded continuously during all diving phases using an HR monitor positioned on the chest belt (Polar T31, Polar Electro Oy, Finland) and interfaced with a dive computer (Galileo SOL, Scubapro Uwatec, California, USA). The data were subsequently downloaded with a Scubapro Uwatec application provided with the dive computer and further stored and analyzed with Microsoft Excel. Computer exported data were HR, depth, and temperature, with a sampling frequency of 0.25 Hz. In all phases, the mean HR was calculated by averaging the values of each temporal phase ($\bar{HR}$). In addition, in both phases, DSC and STD (for the latter excluding the first and last 5 min, hence for approximately 20 min, approximately 80,100 HR samples), linear regressions were assessed. Slope and intercept values were derived from these regressions. In addition, in each phase, the HR root mean square value was calculated. The HR for each individual subject at each depth and in each phase was expressed as a percentage of the own HRpeak as estimated in the exhaustive incremental test to minimize the intrasubject variability. During STD, mean (HR) decreased to 54 ± 12, 51 ± 9 and 49 ± 9 % in −20, −30 and −40 MD respectively137 (Figure 2).

#### 2.2.4 Statistical analysis

The results are expressed as mean ± standard deviation (SD). The Shapiro–Wilk test was applied to verify the homogeneity of the data. A non-parametric Friedman test was run to determine differences in the three slopes and intercepts during DSC and STD between the three depths, followed by a *post-hoc* test (Wilcoxon Signed Rank) to locate statistical differences. Statistical analysis was performed using IBM SPSS, release 25 (IBM, Armonk, NY, USA), with the significance level set at p≤0.05.

## 3 Results

### 3.1 Heart rate analysis

**Figure 3:**
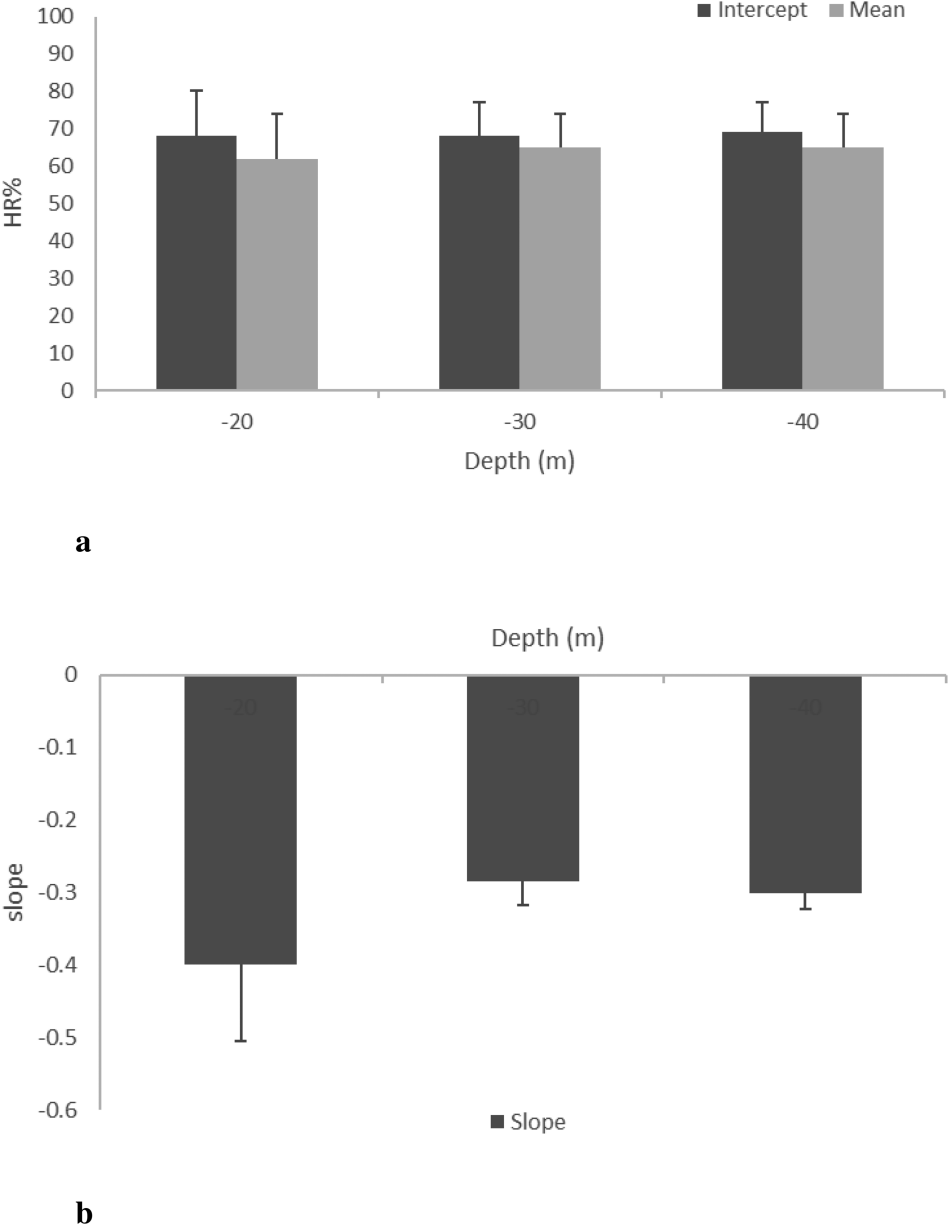
HR mean, intercept (**a**), and slope (**b**) at the three different MDs (20, 30, and 40 m) during DSC (phase 1).

**Figure 4:**
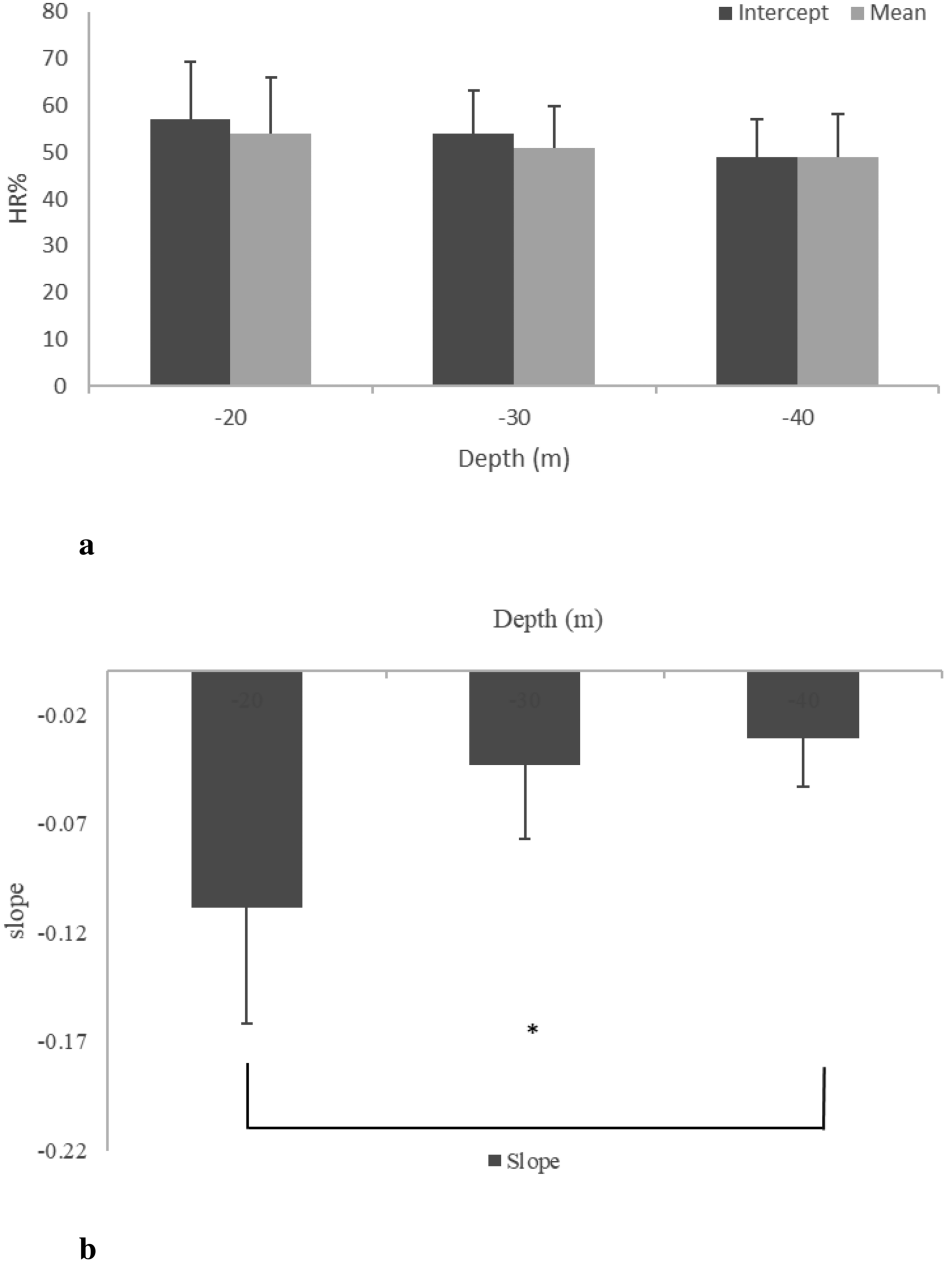
HR mean, intercept (**a**), and slope (**b**) at the three different MDs (20, 30, and 40 m) during STD (phase 2). *p≤0.05 between 20 and 40 m.

The mean ($\bar{HR}$) during DSC was remarkably high (65% HRpeak) with respect to the baseline (52% HRpeak). During STD, the mean ($\bar{HR}$) decreased to 54 ± 12%, 51 ± 9%, and 49 ± 9% at MDs of 20, 30, and 40 m, respectively. After RSF, the mean ($\bar{HR}$) increased at all MDs (20, 30, and 40 m): 61 ± 9%, 60 ± 8%, and 58 ± 9% of HRpeak, respectively (see Figures 3, 4). Intercept and slope parameters obtained by linear regression analysis showed no differences in DSC between MDs (p≤0.05). During STD, slope showed an evident decreasing trend with MD (0.10 ± 0.125 at 20 MD; 0.05 ± 0.064 at 30 MD; 0.03 ± 0.044 at −40 MD). Specifically, a significant difference was found between slopes at 20 and 40 m (p≤0.05). Instead, no differences in slope were observed between 20 and 30 MD and between 30 and 40 MD, as well as between intercepts. Moreover, no differences were found between intercepts (p≤0.05).

## 4 Discussion

This study aimed to study diving HR adjustments in thermal water at different MDs (20, 30, and 40) to investigate the sole hyperbaric effects on cardiac response, reducing thermoregulatory as much as possible. Notably, the huge amount of thermal water in the swimming pool kept the experimental environment constant during all investigated sessions. Each dive was divided into phases to better assess HR changes during the immersion profile. Three periods were identified: descending from 0 to MD (DSC), remaining still at the maximum depth (STD), and the period immediately after resurfacing (RSF). Furthermore, all subjects relaxed during the STD phase for 30 min, avoiding movements for as long as possible. The literature claims that, during diving, the hyperbaric body exposition induces several metabolic and physical adjustments (5, 27, 28). At the cardiac level, HR adjustments are mainly modulated by metabolic responses to the environmental stresses (5, 12, 29). HR values measured at the beginning of diving were the highest recorded in the lab. These increases were likely due to the preparatory phase during which subjects have to wear the wetsuit, set the dive computer, and carry out the predictive mental check (6, 9). During the DSC phase, ($\bar{HR}$) mean data showed an important and clear decrease with quote, mainly attributable to hydrostatic pressure on the glomus caroticum rather than the influence of the trigeminal reflex (7, 14). The latter affects the HR decrease at the face immersion while, with quote changes, the baroreflex induces a rapid HR adjustment, which is almost linear with depth (2). Statistical analysis of the relationship between mean ($\bar{HR}$)% and MD did not reveal differences because of the inter-subject variability. Interestingly, an important result was observed in the second phase of the diving, STD. Note that, at this stage, the diver must reach the assigned maximum depth, hence maintaining an almost vertical position for 30 min, avoiding as many movements as possible after buoyancy apparatus regulation. Unexpectedly, also in this phase, HR% showed a clear slowing, although less pronounced than in DSC. It is important to observe that HR% data, in DSC and STD, fitted the linear model very well (R≥0.09), showing an evident strong relationship between HR% changes and elapsed time. The main result of the present study referred to a significant difference in the mean slope between 20 and 40 MD. To our knowledge, this is a novel datum, and considering the different absolute slope values that respect those during the DSC, it is conceivable that in STD, after reaching the quote MD, a different adaptive physiological mechanism was involved. This result will be deeply discussed below.

### 4.1 DSC and STD HR% adjustments

HR% in DSC and STD was linearized with a mathematical regression and relative mean parameters, intercept and slope, for three MDs, and is assessed and depicted in Figures 3 and 4, respectively. During the DSC, the intercept represents the initial value of HR% at the beginning of the dive, whereas the slope depends on the rate of HR% variation. As evident in Figure 3a, intercept values did not change significantly with MD, and the data are very similar to each other. This result was expected because the stressors influencing HR changes were very similar between MDs in the preparatory phase. Even in Figure 3b, no differences can be observed. It is noteworthy that trigeminal and baroceptor reflexes are the factors influencing the slope. Presumably, both factors act similarly between MDs because the HR effect of the trigeminal reflex is practically constant, whereas the effect of the baroreflex depends on rate of descent, and this was similar for all MDs. As a matter of fact, a standard descent profile was adopted, which was approximately 20 m/min, as suggested by the US Navy manual. In Figure 4b, intercept values show a clear decreasing trend with MD, probably due to the duration of the previous DSC. In other words, a deeper MD implies a longer descent duration that induces a lower HR% at the beginning of STD. This value may change depending on the time required to reach MD. Unexpectedly, no statistical differences occurred with this parameter but this could be accounted for by the fact that every subject approached the end of the descent in a different manner and had successive regulation on the jacket to maintain a stable quote MD. These individual adjustments create a sort of noise on the HR signal. It is noteworthy that to avoid this unwanted HR oscillation, the initial 5 min of HR signals, analogously the final ones, were excluded during the HR processing in the STD phase. In addition, the effect of hyperbaric O^2^, present in this phase, is related to the immersion descent velocity. Finally, in this phase, the subjects changed quotes and pressure in a similar fashion; consequently, the effect on HR% had to be similar. Evidently, in this phase, an increase of dissolved blood O^2^ is also present, similar to what happens during respiration through SCUBA apparatus, to counteract the MD water pressure and allow physiological ventilation. These conditions have been well documented, as hyperoxia influences several regulating molecules, such as angiotensin II, prostaglandins, and adenosine (30), and reactive oxygen species (31), which, in turn, affect cardiovascular functionality, inducing a slowing of the HR (32). HR% slopes with MDs are reported in Figure 4b, and, different to the same parameter in the DSC phase (Figure 3b), a clear decrease with MD increase is evident, suggesting a modification of HR adaptations with the quote and, in turn, water pressure. To our knowledge, this result has not been reported previously, and it reveals a long-term regulation of cardiac frequency in a subject that has no activity at MD. To comment on these long-term physiological changes, some considerations are due. First of all, hyperbaric cardiovascular adaptations have been extensively investigated, mainly in a dry condition; however, many of these results could fit in a wet environment, as in the present study. In this regard, Thomson et al. (32) largely investigated cardiovascular function after a hyperoxic 1 h long breathing of a gas mixture with an O^2^ concentration of 85%. These authors reported that “Hyperoxia reduced the HR (mean −6.7 ± 0.7 beats/min; −10.3 ± 1.0%) (p≤0.001). This was apparent after 5 min and persisted during the hour of hyperoxic exposure.” Moreover, Gole et al. (33) monitoring HR after breathing a high concentration of O^2^ for 45 min reported a clear slowing during hyperbaric conditions and a restoration of HR 10 min from the end onward. Unfortunately, these authors did not measure parameters in hyperbarism frequently and it was impossible to know the HR kinetics from normobaric to hyperbaric. Based on these results, it is possible to affirm that in the dry, the passage of O^2^ breathing between normobaric to hyperbaric, and *vice versa*, induces HR changes with a time evolution in the range of 510 min. Hence, HR stabilized at the new value. Again, at MD, the water pressure compresses all body tissues thus pushing the blood to the inner part of the body (34). Although this blood rearrangement is well documented in breath-holding diving, it is presumable that it is also present in SCUBA diving in several parts of the body. In particular, the divers in this study were in a vertical body position at MD, performing very little muscular activity during the STD phase. In this setting, all body parts except the chest were compressed. As a result, blood shifted to the central regions of the body, increasing venous return to the heart. This blood shift leads to reflex bradycardia, causing the heart to slow in response to the increased volume load (35). Unlike hyperoxia, the blood shift effect from distal to central parts of the body continues to influence the body throughout the duration of the dive, providing a more comprehensive explanation for the sustained bradycardia observed in divers (35). In summary, hyperoxia leads to rapid initial changes in cardiorespiratory physiology. At the same time, the squeezing effect sustains a long-term adaptation during diving, explaining the phenomenon behind the mechanisms that induce bradycardia in immersed individuals (36).

## 5 Conclusions

Studies of diving in thermal water allow for the reduction in the thermoregulation effect in HR adjustments and highlight the hyperbaric contribution to the cardiovascular system. The present study emphasized two different HR physiological adjustments. First, during the DSC, a rapid HR decrease is recognized. Second, while staying at MD, the blood redistribution requires a different adjustment. The latter is depth-dependent because of the residual cardiac variability. The present data highlighted that the HR needed to counteract the diving activity that, in this controlled and comfortable condition, was very important. Presumably, operative diving at work can result in a higher HR because of the low temperature and workload. All of this underlines the importance of a careful medical and cardiological evaluation, which should be mandatory for professional divers. In perspective, blood pressure measurements should be implemented to investigate the whole cardiovascular response besides HR.

## Data Availability

The original contributions presented in the study are included in the article/Supplementary Material, further inquiries can be directed to the corresponding author.
