# Supplementary material for: Hyperbaric effects on heart rate in professional SCUBA divers in thermal water
Source: Front Sports Act Living. 2024 Sep 30;6:1429732. doi: 10.3389/fspor.2024.1429732 (PMC11471545; doi:10.3389/fspor.2024.1429732)
Supplement: Supplementary file 1 [file Datasheet1.pdf]

Supplementary Material

1 SUPPLEMENTARY DATA

2 SUPPLEMENTARY TABLES AND FIGURES

2.1 Tables and Figures

Divers characteristics	Mean	SD
Age (y)	48	7
Height (m)	1.71	0.12
Weight (kg)	76	12
HRrest (bpm*min^{-1})	82	8
HRpeak (bpm*min^{-1})	164	4
VO2max (ml/kg/min)	30	5
VT1 % (respect VO_2max)	57	4
VT2 % (respect VO_2max)	68	3

Table S1. Divers physiological and anthropometrical characteristics.

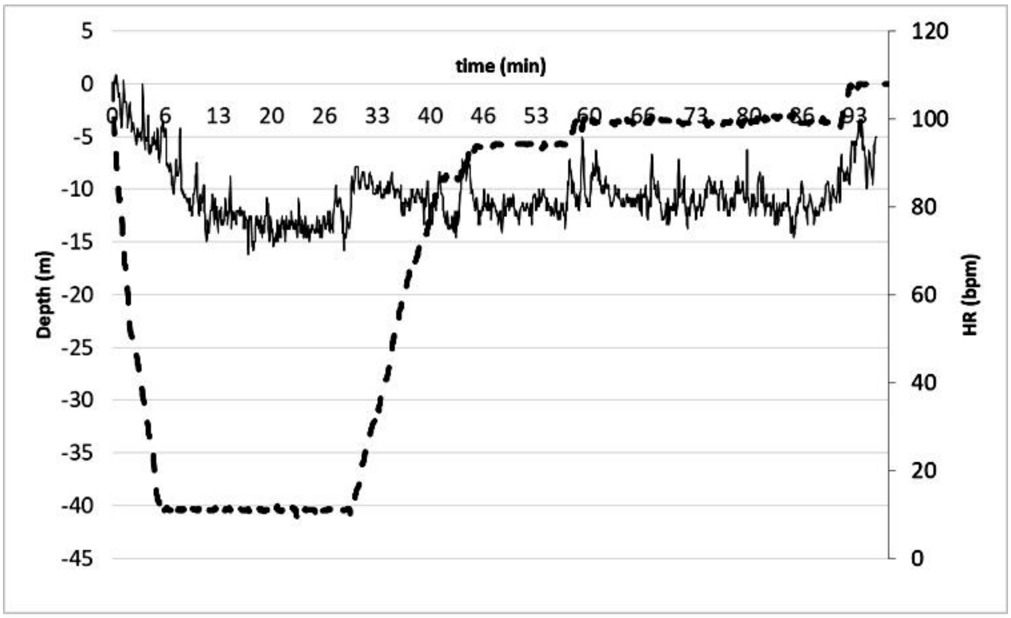

Figure S1. Dotted line represents the instantaneously depth, while continuous the HR. As evident HR was remarkable high during the pre-dive and dropped rapidly during the descent phase, then decreased slightly during the steady phase and finally returned to the pre-dive values in the resurfacing. Note the decompression stops before resurfacing.

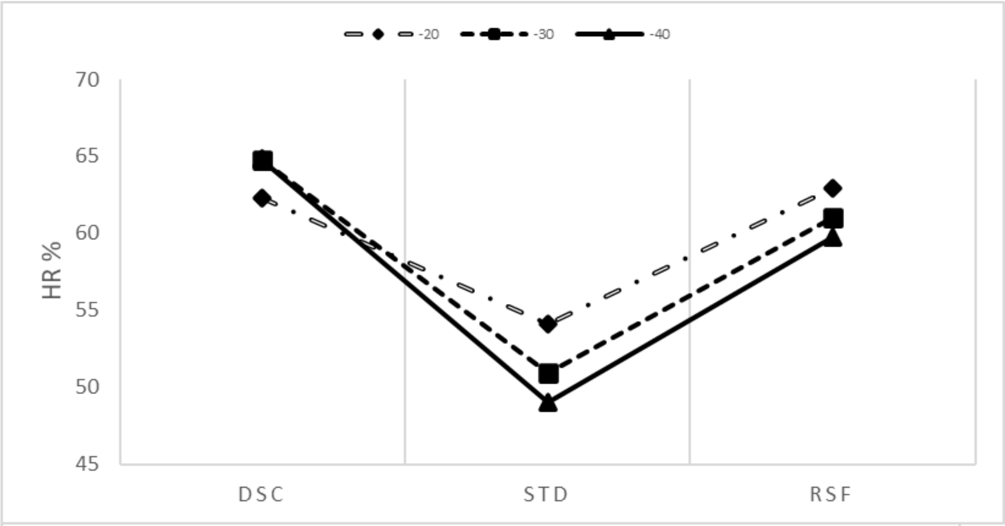

Figure S2. $\overline{HR}$ % (scaled by HR peak) trend in all depth (-20, -30, -40) during descent (DSC), dive (STD) and after resurfacing (RSF). Intercept and slope parameters obtained by linear regression analysis showed no differences in DSC among MD ($p \leq .05$).

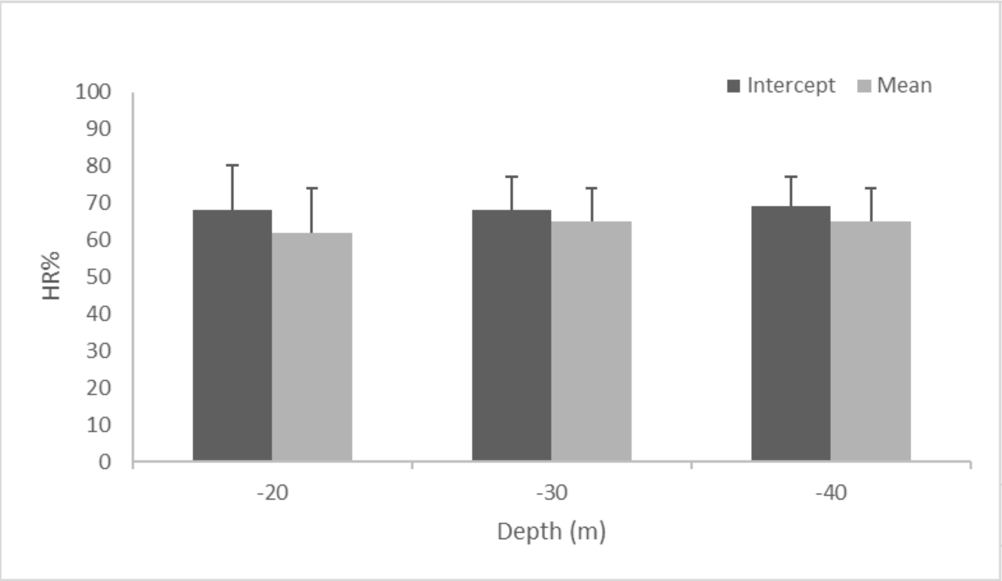

Figure 3a.

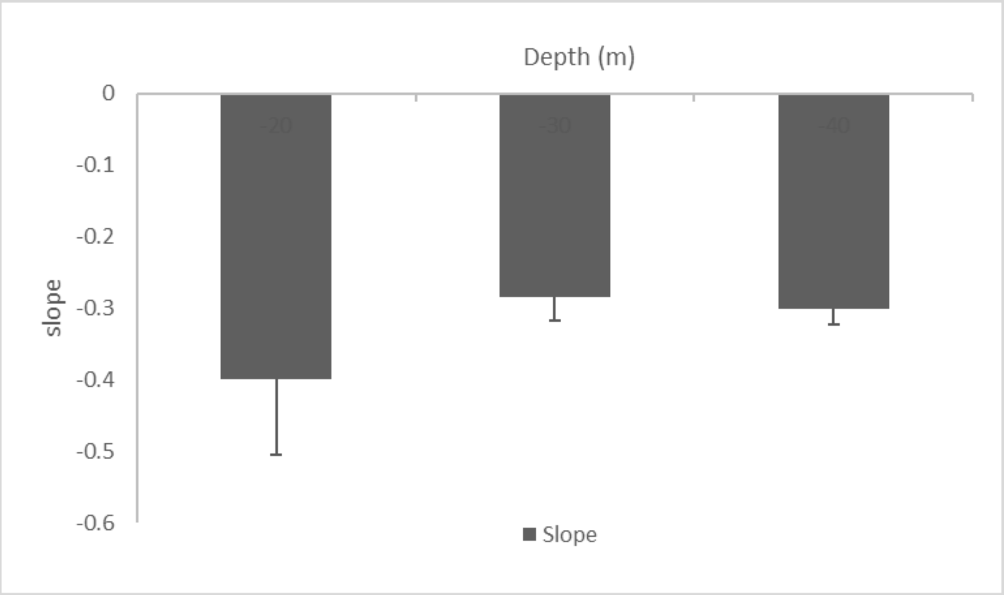

Figure 3b.

Figure 3. HR mean, intercept (panel **A**) and slope (panel **B**) in the three different MD (-20, -30, -40 m) during DSC (phase 1).

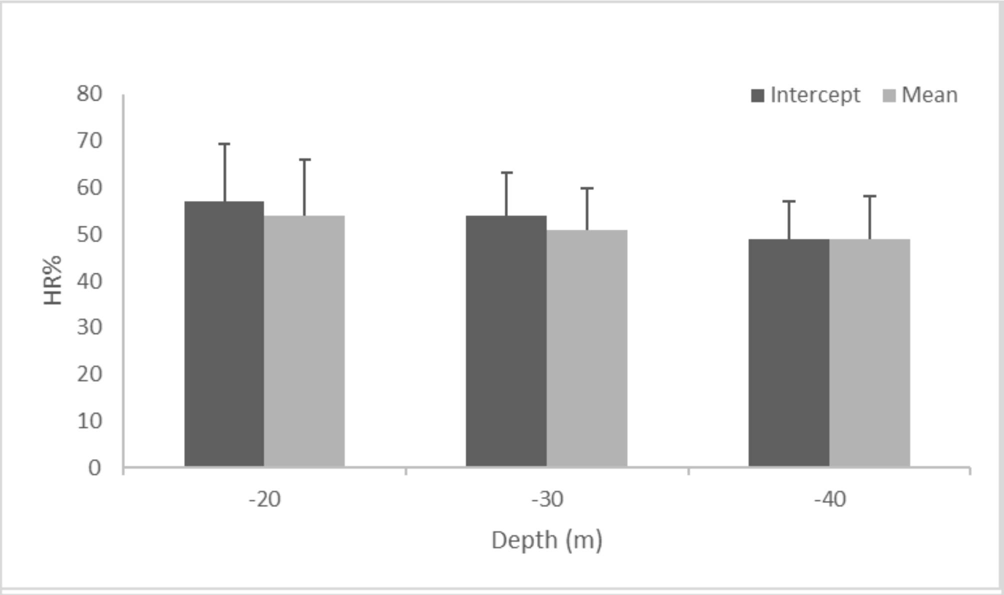

Figure 4a.

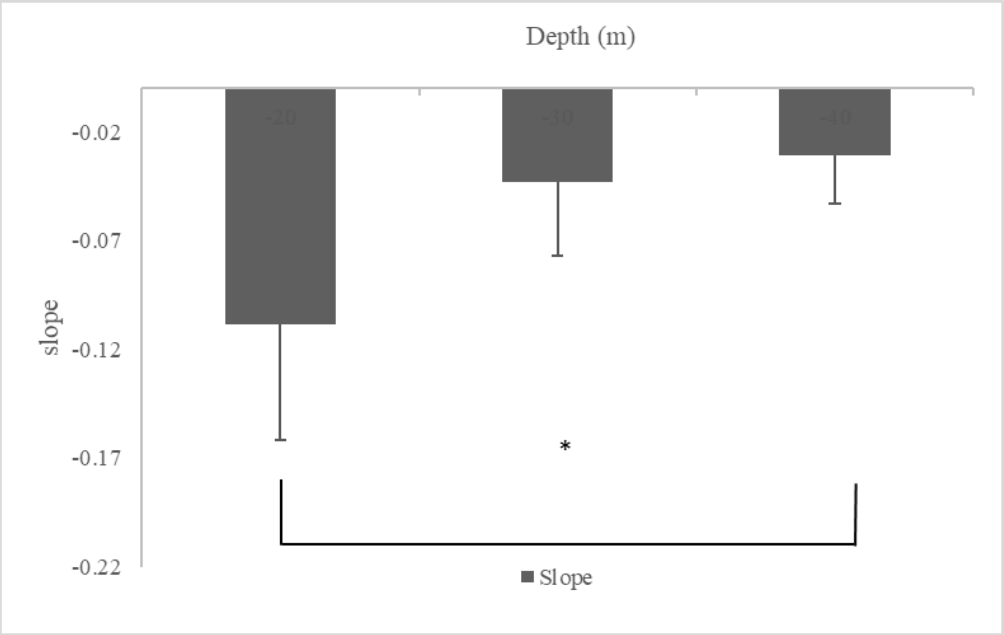

Figure 4b.

Figure 4. HR mean, intercept (panel **A**) and slope (panel **B**) in the three different MD (-20, -30, -40 m) during STD (phase 2). $*p \leq .05$ between -20 and -40 m.
